# Pan-cancer analysis identifies venous thromboembolism-related genes F3, PLAT, and C1S as potential prognostic biomarkers for glioblastoma and lower grade glioma

**DOI:** 10.1186/s43556-024-00197-9

**Published:** 2024-08-24

**Authors:** Jing Zhang, Qian Zhao, Yun Du, Wannan Wang, Cuiqing Liu

**Affiliations:** 1 Department of Radiology, The First Affiliated Hospital of Jinan University, 510630 Guangzhou, China; 2 Present Address: MOE Key Laboratory of Tumor Molecular Biology and Key Laboratory of Functional Protein Research of Guangdong Higher Education Institutes, College of Life Science and Technology, Institute of Life and Health Engineering, Jinan University, 510632 Guangzhou, China; 3 Present Address: Department of Nursing, The First Affiliated Hospital of Jinan University, 510630 Guangzhou, China; 4 Department of Surgery, The First Affiliated Hospital of Jinan University, 510630 Guangzhou, China

**Keywords:** Venous thromboembolism, Coagulation, Complement, Pan-cancer, Tumor immune microenvironment

## Abstract

Venous thromboembolism (VTE) is a prevalent complication among patients with cancer, contributing significantly to morbidity and mortality. However, the relationship between VTE-related genes (VRGs) and their potential impact on prognosis, immune response, and therapeutic targets in various cancer types remains unclear. Based on the coagulation and complement pathways, we identified hub VRGs that play a role in regulating the immune response in cancer. Specifically, coagulation factor III (F3), plasminogen activator (PLAT) and complement C1s (C1S) were identified as genes that exhibit high expression levels, positively correlating with tumor stemness and copy number variations, while inversely correlating with methylation levels, in particular cancer types. Pan-cancer survival analysis revealed detrimental effects of these VRGs in several cancer types, notably in glioblastoma and lower grade glioma (GMBLGG). Further analysis using receiver operating characteristic (ROC) curves demonstrated a high accuracy of F3, PLAT and C1S in predicting outcomes in GBMLGG, with area under the curve (AUC) values ranging from 0.78 to 0.9. Validation of the prognostic value of these three genes in GMBLGG was conducted using an independent Gene Expression Omnibus (GEO) dataset. Additionally, gene–drug association analysis identified ciclosporin, ouabain and 6- mercaptopurine, which all exhibit immunosuppressive properties, as potential therapeutic options for tumor patients exhibiting high F3, PLAT or C1S expression, respectively. In summary, our findings provide a bioinformatics perspective on VRGs in pan-cancer, highlighting the pivotal roles of F3, PLAT and C1S, which could potentially be therapeutically exploited and targeted in several cancers, especially in GBMLGG.

**Supplementary Information:**

The online version contains supplementary material available at 10.1186/s43556-024-00197-9.

## Introduction

According to the Global cancer statistics 2022, cancer remains one of the leading causes of death worldwide, with 9.7 million deaths and 20 million new cases reported [1]. Patients with cancer face an increased risk of venous thromboembolism (VTE), which significantly impacts morbidity and survival rates [2]. VTE incidence in cancer varies widely, ranging from 2 to 14%, with pancreatic, lung, and stomach cancers posing the highest risk [3]. The incidence of VTE in cancer patients varies depending on the cancer type and individual patient-related and cancer treatment factors.

The pathophysiology of VTE is complex and multifactorial [4], involving coagulation and complement activation as key risk factors [5, 6]. The coagulation pathway, pivotal in blood clots formation [6], begins with tissue factor (TF) interacting with its cofactor, blood coagulation factor VII (FVII), resulting in the conversion of prothrombin into thrombin [7]. This, in turn, triggers a cascade of reactions involving platelets, other coagulation factors and the complement pathway, collectively promoting the development of VTE. Similarly, the complement pathway, a component of the innate immune system, plays a pivotal role in the pathogenesis of VTE [5]. Within the tumor microenvironment (TME), the complement system possess diverse and intricate functions, such as elimination of tumor cells coated with antibodies, promotion of chronic inflammation at the tumor site, and modulation of T-cell response [8]. Upon activation, the complement system triggers inflammation, which enhances the formation of blood clots by upregulating the expression of tissue factor on monocytes and endothelial cells [9]. Moreover, dysregulation of the coagulation and complement pathways can lead to a prothrombotic state, increasing the risk of VTE across all stages of cancer [7].

Recent advancements in genomics have identified several venous thromboembolism-related genes (VRGs), link to molecular mechanisms underlying VTE. For instance, mutations in genes encoding coagulation factors such as coagulation factor II (F2) and coagulation factor V (F5) have been linked to an increased susceptibility to VTE [10]. The complement factor (C3bBbP) is associated with an increased risk of provoked VTE in nested case–control investigations [11]. Additionally, a large population-based study revealed that individuals with higher plasma levels of complement C3 have a greater likelihood of developing VTE compared to those with lower levels [12]. However, given the limited number of patient samples examined in previous studies, the biomedical significance of VRG expression in cancer remains incompletely understood. Notably, a crucial question that arises is whether the expression of VRGs can effectively characterize the tumor heterogeneity within a specific cancer type and serve as a meaningful dimension for patient stratification. To address this gap in knowledge, it is imperative to conduct a comprehensive analysis across large patient cohorts.

This study identifies candidate VRGs associated with cancer-related VTE, focusing on the coagulation and complement pathways. We comprehensively analyze these genes across various cancers, evaluating their prognostic significance, expression patterns, interaction with the TME, genetic alterations and drug sensitivity. These insights enhance our understanding of VRGs, potentially refining of therapeutic strategies and prognostic predictions in cancer treatment.

## Results

### Recognition of key VRGs

**Fig. 1:**
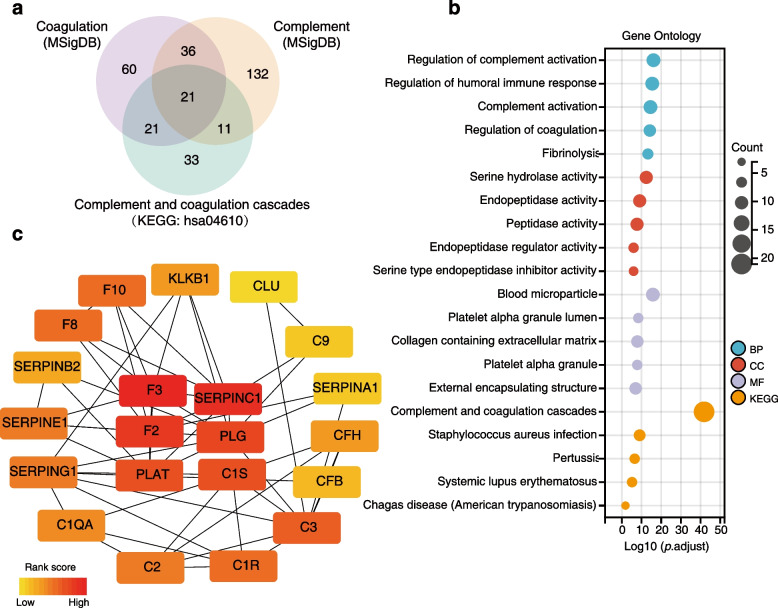
Identification of VTE-related genes from complement and coagulation pathways. **a** Venn diagram displaying the overlaps of genes from complement and coagulation pathways in the MSigDB and KEGG databases. **b** GO and KEGG enrichment analysis of the 21 VTE-related genes. (c) The top six genes were optimized by PPI networks using cytoHubba

To identify genes related to VTE, we overlapped genes from the complement and coagulation pathways acquired from Molecular Signatures Database (MSigDB) and Kyoto Encyclopedia of Genes and Genomes (KEGG) database, and a total of 21 candidate genes were obtained (Fig. 1a). Gene Ontology (GO) and KEGG enrichment analyses showed that these 21 VRGs also regulate immune response in addition to complement and coagulation activation (Fig. 1b). To further screen out key VRGs, a protein–protein interaction (PPI) network was constructed, and the maximum neighborhood component (MNC) algorithm was utilized to identify hub genes. Based on their rank scores, we identified coagulation factor III (F3), serpin family C member 1 (SERPINC1), F2, plasminogen (PLG), plasminogen activator (PLAT) and complement C1s (C1S), as the top 6 genes (Fig. 1c).

### Association between VRGs and immune microenvironment

**Fig. 2:**
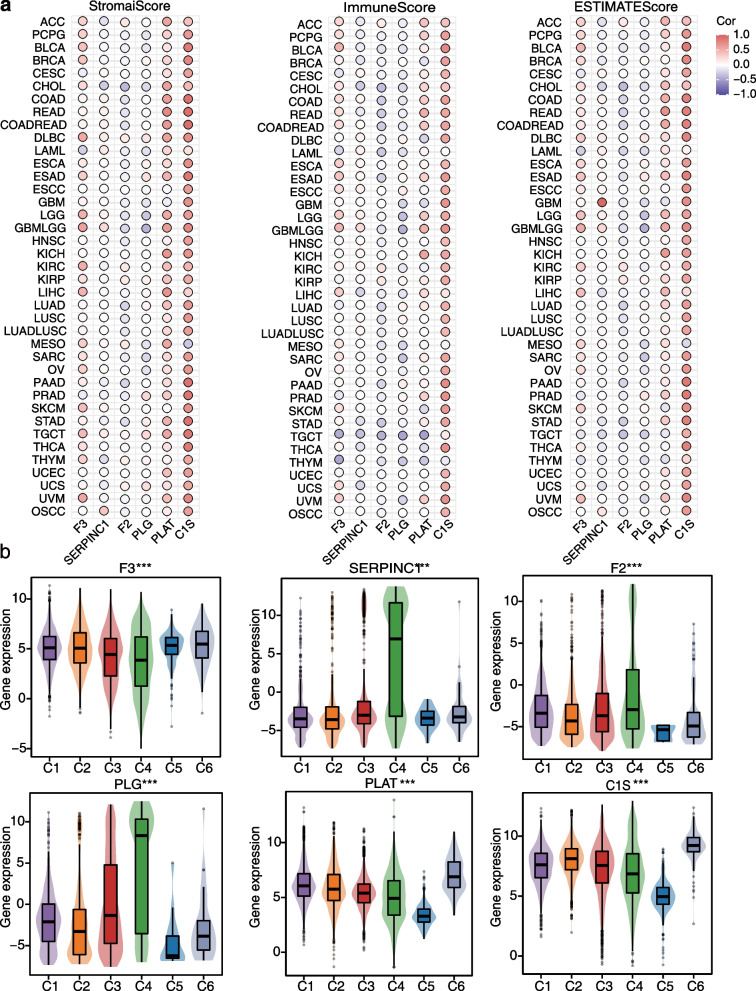
The relationship between VRGs and immune scores and subtypes. **a** The correlation between VRGs and stromal score, immune score, and ESTIMATE score across various cancers. **b** VRGs mRNA expression in six immune subtypes. C1 refers to wound healing, C2 refers to IFN-gamma dominant, C3 refers to inflammatory, C4 refers to lymphocyte depleted, C5 refers to immunological quiet, and C6 refers to TGF-beta dominant. Mean ± SD, one-way ANOVA, ***p < 0.001

Evidence from our functional enrichment analysis indicates that VRGs regulate the immune response. To further explore this, we first assessed the relationship between VRG expression and immune scores, immune subtypes and immune infiltration across different cancers. In the majority of cancer types, F3, PLAT, and C1S exhibited significantly stronger positive correlations with stromal, immune and ESTIMATE scores compared to F2, PLG, and SERPINC1 (Fig. 2a). Furthermore, employing the xCell method to assess VRGs and immune cell infiltration (Fig. S1), we observed that F3, PLAT, and C1S displayed a positive correlation with a wide array of immune cells across diverse cancer types, consistent with the observed patterns in ESTIMATE scores. This suggests that F3, PLAT, and C1S may enhance immune responses within the tumor microenvironment.

Given the predictive value of immune subtype analysis in cancer treatment [13], we explored the relationship between VRG expression and immune subtypes in pan-cancer. Our analysis indicated that F3, PLAT, and C1S exhibited highest expression in subtype C6, characterized as “TGF-beta dominant”, whereas the other three VRGs showed the highest expression in subtype C4, known as “lymphocyte depleted” (Fig. 2b). These findings underscore the potential of VRGs to influence immune response in cancer, potentially impacting disease progression and therapeutics outcomes.

### Relationship between VRGs and tumor stemness and heterogeneity

**Fig. 3:**
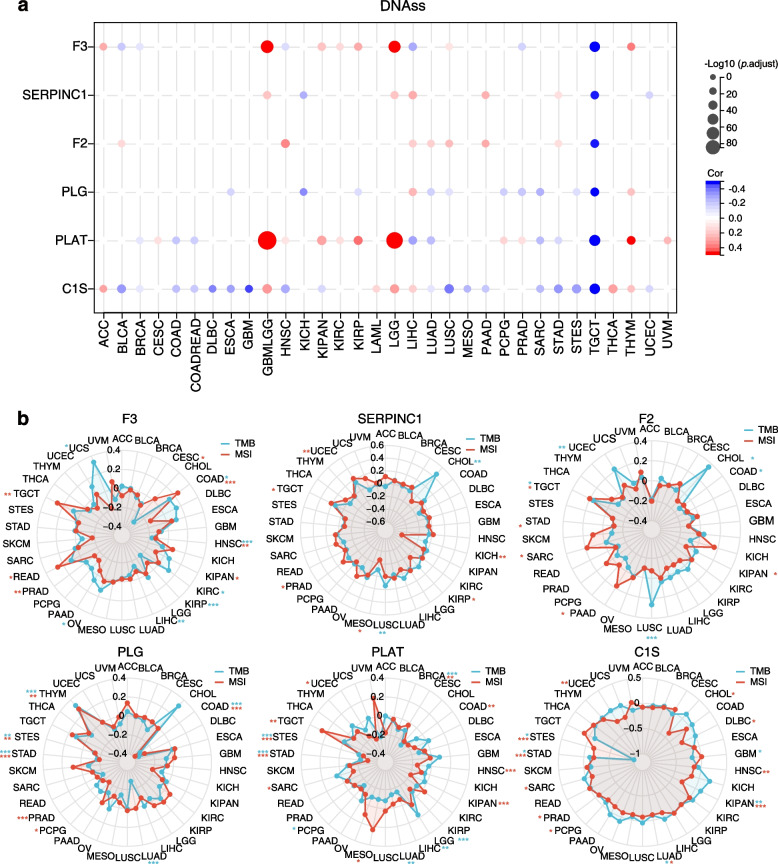
Correlation between VRG expression and tumor stemness and heterogeneity in pan-cancer. **a** Buble pltos showing the correlation between VRGs and DNAss in different cancers. **b** Radar diagram depicting the correlation between VRGs and TMB/MSI. T-test, *p < 0.05, **p < 0.01, ***p < 0.001

Tumor stemness and heterogeneity significantly influence the cancer prognosis and treatment outcomes [14–16]. To explore their connection with VRGs, we first examined the relationship between the expression levels of VRGs and DNA stemness scores (DNAss) across various cancers. Our analysis revealed a positive correlation between VRG expression and DNAss in specific cancers, particularly, F3, PLAT, and C1S and DNAss in glioblastoma and lower grade glioma (GBMLGG) (Fig. 3a), suggesting a potential role of these genes in promoting tumor stemness in these specific cancers. Next, we assessed the correlation between VRGs and tumor heterogeneity using tumor mutational burden (TMB) and microsatellite instability (MSI) (Fig. 3b). Our radar plots revealed that F3, SERPINC1, F2, PLG, and PLAT exhibited the highest positive correlation with TMB in uterine carcinosarcoma (UCS), cholangiocarcinoma (CHOL), CHOL, thymoma (THYM) and LGG, respectively. These genes also displayed a strong correlation with MSI in rectum adenocarcinoma (READ), testicular germ cell tumors (TGCT), TGCT, THYM and TGCT respectively. These findings indicate that the aforementioned VRGs may contribute to tumor heterogeneity in these specific cancer types. Additionally, C1S displayed relatively lower correlation with both TMB and MSI. Collectively, these findings suggest that VRGs have the potential to play crucial roles in promoting tumor stemness across specific cancer types, meanwhile highlighting the complex relationship between VRGs and tumor heterogeneity.

### The genetic alterations and methylation of VRGs

**Fig. 4:**
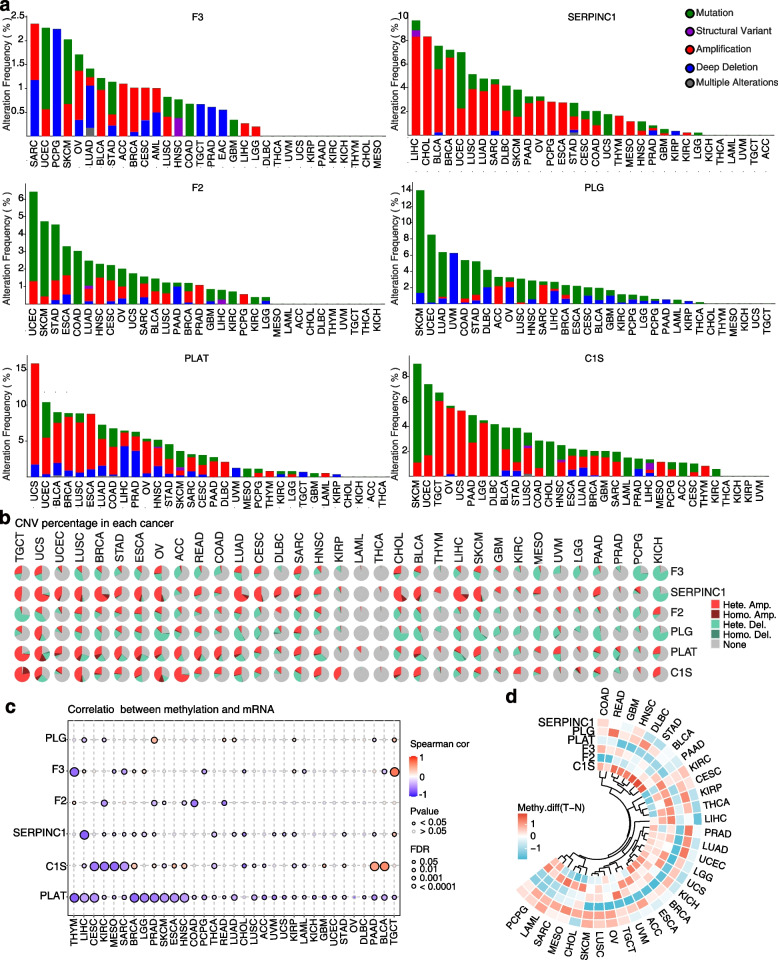
The genetic alterations and methylation of VRGs. **a** Genetic alteration frequency of VRGs. Green indicates mutation, purple indicates structural variant, red indicates amplification, blue indicates deep deletion, and gray indicates multiple alterations. **b** The statistics for deletion and amplification of heterozygous and homozygous CNV of VRGs. **c** Correlations between DNA methylation and VRGs. **d** The degree of methylation variation observed in paired tumor and adjacent normal tissue samples

Genomic and epigenetic alterations play critical roles in tumor development and immune response [17]. We investigated genetic alterations of VRGs across different cancers using the cBioPortal portal, revealing varying frequencies of mutations, amplifications, deletions, and other alterations (Fig. 4a). The overall alteration frequency ranged from 0% to 15.8%, with relatively high variation frequency observed in PLG, SERPINC1, and PLAT. Amplification was found to be the most prevalent alteration type for SERPINC1 and PLAT, with high amplification frequencies notably observed in UCS, liver hepatocellular carcinoma (LIHC), CHOL, and breast invasive carcinoma (BRCA). Additionally, skin cutaneous melanoma (SKCM) and uterine corpus endometrial carcinoma (UCEC) showed relatively high mutation frequencies for F2, PLG, and C1S. However, certain cancers such as kidney chromophobe (KICH), thyroid Cancer (THCA), and uveal melanoma (UVM) exhibited rare VRGs genetic alterations. We also examined the prevalence of copy number variation (CNV) in VRGs across different cancers using the Gene Set Cancer Analysis (GSCA) database (Fig. 4b). Heterozygous CNV amplification were widespread, especially in TGCT, UCS, UCEC, lung squamous cell carcinoma (LUSC), BRCA, stomach adenocarcinoma (STAD), esophageal carcinoma (ESCA), ovarian cancer (OV), and adrenocortical carcinoma (ACC). Furthermore, heterozygous CNV deletions emerged as the second most frequent occurrence, particularly for F2 and PLG.

To assess the potential impact of DNA methylation on the mRNA expression of VRGs, correlation analyses were conducted (Fig. 4c). We found that VRGs showed a negative correlation with the degree of methylation in most cancers, with some exceptions, such as C1S in pancreatic cancer (PAAD) and Bladder Cancer (BLCA), F3 in TGCT, which exhibited a positive correlation between the expression of these genes and the methylation levels. Comparing methylation patterns between tumor and normal groups revealed diverse VRG methylation profiles across different cancers. Notably, relatively lower methylation levels for SERPINC1 and PLAT were observed in most cancers than that in normal groups (Fig. 4d). These findings underscore the complex genetic alterations and methylation patterns of VRGs across different cancers, suggesting their potential roles in modulating gene expression and influencing cancer progression.

### Expression and prognostic value of VRGs in *pan*-*cancer*

**Fig. 5:**
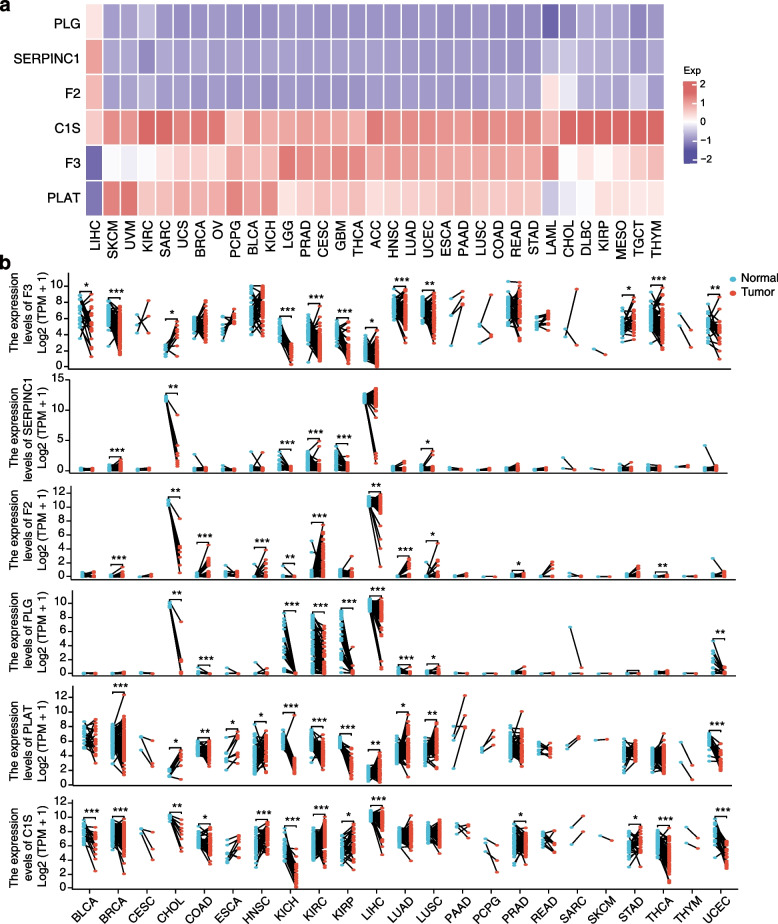
VRG expression levels in pan-cancer. **a** VRG expression levels across various cancers. **b** The mRNA expression of F3, SERPINC1, F2, PLG, C1S, and PLAT were analyzed in paired cancer and adjacent normal tissue samples. Wilcoxon signed rank test, *p < 0.05, **p < 0.01, ***p < 0.001

**Fig. 6:**
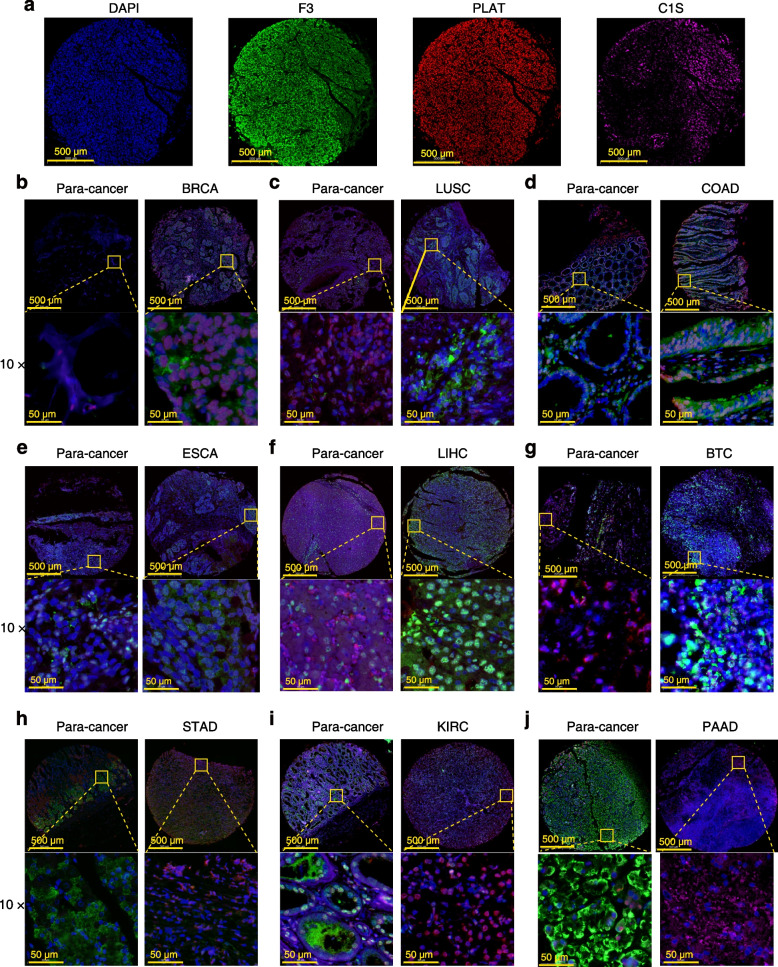
Multiplex immunofluorescence staining of F3, PLAT and C1S was performed in pan-cancer and the corresponding para-cancer tissues. **a** Representative images of DAPI, F3, PLAT and C1S staining. Blue, DAPI-stained nucleus; green, F3; red, PLAT; pink, C1S. Scare bar, 500 µm. **b** Breast invasive carcinoma (BRCA), *n* = 5. **c** Lung squamous cell carcinoma (LUSC), *n* = 12. **d** Colon adenocarcinoma (COAD), *n* = 6. **e** Esophageal carcinoma (ESCA), *n* = 6. **f** Liver hepatocellular carcinoma (LIHC), *n* = 6. **g** Extrahepatic biliary tract cancer (BTC), *n* = 5. **h** Stomach adenocarcinoma (STAD), *n* = 6. **i** Kidney renal clear cell carcinoma (KIRC), *n* = 6. **j** Pancreatic adenocarcinoma (PAAD), *n* = 6

To investigate the differential expression of VRGs across different cancer types, we first examined their relative mRNA expression levels using data from The Cancer Genome Atlas (TCGA). Our analysis identified that C1S, F3, and PLAT as genes with notably high expression, while PLG, SERPINC1, and F2 exhibited lower expression levels across most cancers (Fig. 5a). Comparing VRG expression between paired cancer and para-cancer tissue samples revealed downregulation in KICH, CHOL, kidney Clear Cell Carcinoma (KIRC), kidney papillary cell carcinoma (KIRP), and UCEC, and upregulation in LUSC (Fig. 5b). Notably, compared to paired normal tissues, lower PLG expression was observed in eight cancer types, with the exception of LUSC, which showed high expression of PLG. Conversely, higher F2 and F3 expressions appeared most frequently in different cancers. Using multiplex immunofluorescence staining, we confirmed elevated expression of F3, PLAT and C1S in many cancer tissues as compared to paired para-cancerous tissues. As indicated in Fig. 6, the numbers of F3-positive cells were elevated in BRCA, LUSC, COAD, LIHC and extrahepatic biliary tract cancer (BTC), PLAT-positive cells were elevated in STAD, KIRC and PAAD, and C1S-positive cells were elevated in the BRCA, and STAD, in comparison with their corresponding para-cancerous tissues.

**Fig. 7:**
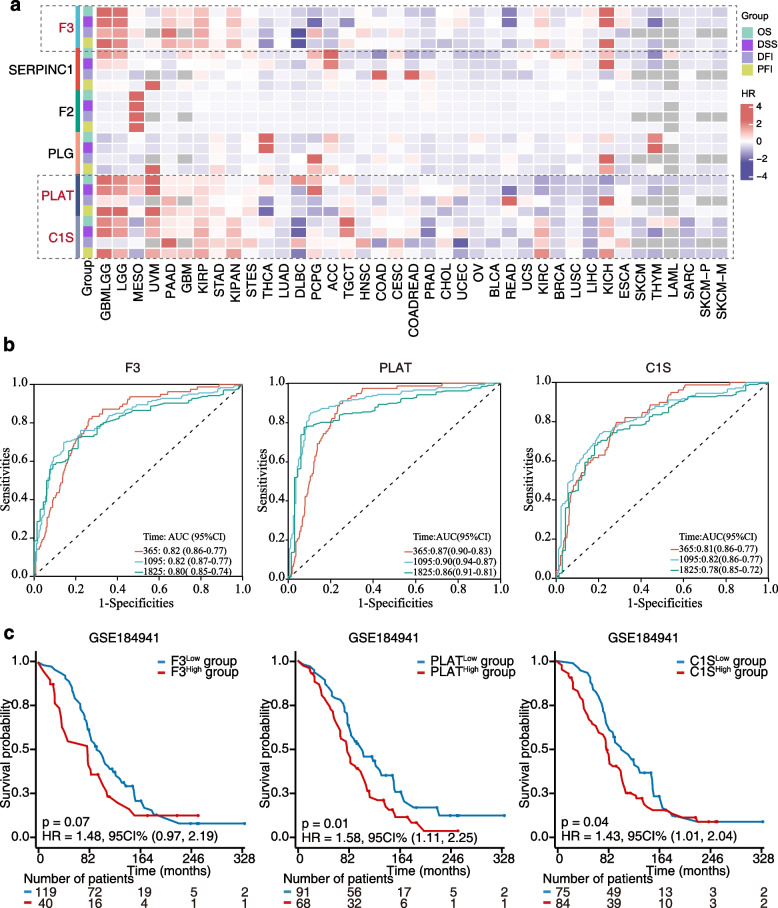
Prognostic values of F3, PLAT and C1S (**a**) Heatmap showing the correlation between VRGs and OS, DSS, DFI, and PFI in TCGA. **b** Diagnostic ROC curves for F3, PLAT and C1S in GBMLGG. **c** Overall survival curves of F3, PLAT and C1S were analyzed in a GMBLGG GEO dataset

Additionally, we investigated the relationship between VRG mRNA expression and patient prognosis, including overall survival (OS), disease-specific survival (DSS), disease-free interval (DFI), and progression-free interval (PFI) values using TCGA data. Our analysis highlighted F3, PLAT and C1S as genes associated with poorer prognosis across various cancers (Fig. 7a). Forest maps illustrating OS for F3, PLAT and C1S underscored their significant roles, particularly in GBMLGG (Fig. S2-S4). Furthermore, receiver operating characteristic (ROC) curves demonstrated the diagnostic accuracy of these three VRGs in predicting outcomes in GBMLGG tissues, with high area under the curve (AUC) values ranging from 0.78 to 0.9 for 1-, 3-, and 5-year predictions (Fig. 7b). Considering the substantial prognostic impact of the three key VRGs, we validated their prognostic values using a GBMLGG dataset from the Gene Expression Omnibus (GEO) (Fig. 7c). Our results indicated that individuals with high F3 (HR = 1.48, 95%CI = 0.97–2.19, *p* = 0.07), PLAT (HR = 1.58, 95%CI = 1.11–2.25, *p* = 0.01) or C1S (HR = 1.43, 95%CI = 1.01–2.04, *p* = 0.04) expression experienced significantly poorer prognosis, which was consistent with the observations in the TCGA dataset (Fig. 7d). These findings suggest the crucial role of VRG expression in predicting tumor prognosis.

### Drug sensitivity analysis of VRGs

**Fig. 8:**
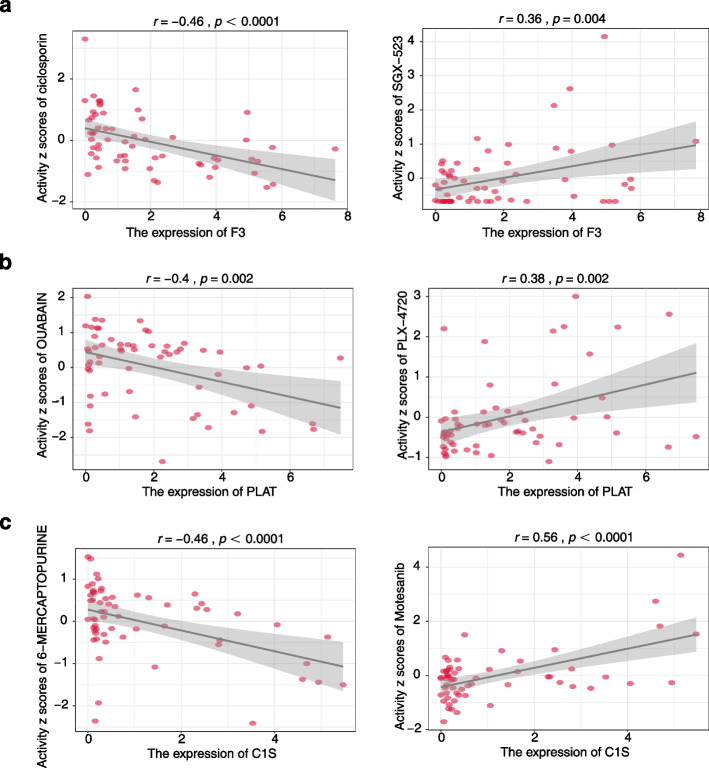
Drug sensitivity analysis of VRGs. Scatter plots and fitting curves showing positive and negative correlations between the sensitivity of representative drugs and the expression of F3 (**a**), PLAT (**b**), and C1S (**c**)

Given the substantial negative impact that F3, PLAT, and C1S have on prognosis, targeting these genes hold promise as a therapeutic strategy. We further explored their potential clinical applications by assessing drug sensitivity using the CellMiner™ database. Notably, we observed that the drug sensitivity of ciclosporin (*r* = -0.46, *p* < 0.0001) and SGX-523 (*r* = 0.36, *p* = 0.004) displayed the most negative and positive correlation with F3, respectively (Fig. 8a). For PLAT, ouabain (*r* = -0.4, *p* = 0.002) demonstrated the highest negative correlation, while PLX-4720 (*r* = 0.38, *p* = 0.002) exhibited the strongest positive correlation (Fig. 8b). Lastly, for C1S, 6-mercaptopurine (*r* = -0.46, *p* < 0.0001) displayed the most negative correlation with C1S, whereas motesanib (*r* = 0.56, *p* < 0.0001) showed the highest positive correlation (Fig. 8c). These results suggest that patients with tumor expressing high expression of F3, PLAT or C1S may benefit from ciclosporin, ouabain, and 6-mercaptopurine, respectively.

## Discussion

The study explored the potential role of VRGs in pan-cancer (Fig. S5). We identified six hub VRGs including F3, SERPINC1, F2, PLG, C1S, and PLAT based on coagulation and complement pathways annotated in the MSigDB and KEGG databases. Previous studies have individually investigated these genes in relation to tumors and VTE. F3, encoding coagulation factor III, a high-affinity receptor that induce the activation of coagulation factor VII, thereby increasing VTE risk in various tumor types [18]. In GBM cells, F3 could promote proliferation and orchestrates oncogenic TME remodeling by activating both tumor-autonomous signaling and extrinsic coagulation pathways [19]. SERPINC1 encodes antithrombin III (ATIII), a serine protease inhibitor whose specific role in tumor biology is not yet clear [20]. F2 encodes prothrombin, mutations in which are linked to an elevated incidence rate of VTE [21]. PLG encodes plasminogen, pivotal in hemostasis and involved in multiple biological processes during cancer proliferation and dissemination [22, 23]. PLAT encodes tissue-type plasminogen activator and contributes to cancer cell migration and tissue remodeling [24]. Yamashita et al. reported that PLAT, regulated by miR-340, serves as a pivotal molecule in promoting the malignancy of glioma-initiating cells [25]. C1S encodes a serine protease crucial to the classic complement pathway, potentially promoting tumor progression through both complement cascade-dependent and -independent manners [26]. Notably, our study provides the first evidence that F3, PLAT, and C1S consistently exhibited elevated expression levels in the majority of tumors compared to SERPINC1, F2, and PLG. Furthermore, our multiplex immunofluorescence staining confirmed the increased protein levels of C1S, F3, and PLAT in cancer tissues compared to their paired para-cancerous tissues. Interestingly, most VRGs exhibited a positive correlation with CNV frequency and a negative correlation with t methylation levels. These variations in CNV and methylation levels likely contribute to observed differences in VRG expression levels in tumors [8].

Tumor stemness, which refers to the capability of cancer cells to self-renew, is linked to tumor initiation, progression, metastasis, therapeutic resistance, and immune escape [27]. Our findings reveal that F3, PLAT, and C1S exhibit positive correlations with tumor stemness in certain cancer types, suggesting that these VRGs may exert detrimental effects in those specific cancer types. Furthermore, our prognosis analysis supports that F3, PLAT, and C1S had potential diagnostic value, particularly in GBMLGG. GBMLGG, which comprises glioblastoma and low-grade glioma, is well-recognized as a high-risk tumor type for VTE [28]. Consistent with our results, Saidak et al. reported a high expression of F3 in GBM and observed a positive correlation between F3 expression and the risk of VTE [29]. Notably, our study further emphasizes the remarkable predictive accuracy of F3, PLAT, and C1S in forecasting GBMLGG outcomes, with AUC values ranging from 0.78 to 0.9. These findings underscore the potential of these genes as prognostic biomarkers and emphasize their importance in disease management.

Interestingly, our research reveals that tumors expressing high levels of F3, PLAT, and C1S are notably characterized by a “hot” immune microenvironment. Specifically, these three genes demonstrate a robust positive correlation with immune scores and infiltrating immune cells, indicating their potential involvement in immune activation. This finding is consistent with recent studies highlighting the association between coagulation pathways and TME across multiple cancer types [29]. Thorsson et al. identified six stable and reproducible immune subtypes, C1-C6, associated with prognostic, genetic and immune modulatory alterations, which play a key role in predicting disease prognosis [13]. Our study discovered that F3, PLAT and C1S exhibit the highest expression in subtype C6, known as “TGF-beta dominant,” which is characterized by elevated TGF-beta signaling that may suppress immune response and promote tumor growth. Conversely, SERPINC1, F2 and PLG, the other three VRGs, show the highest expression in subtype C4, known as “lymphocyte depleted,” which is marked by low lymphocyte and high macrophage infiltration, along with genes involved in epithelial-to-mesenchymal transition and angiogenesis. Notably, both C4 and C6 subtypes are associated with an immunosuppressed TME, predicting a poorer prognosis. Herein lies an apparent paradox: while F3, PLAT, and C1S correlate positively with immune scores and infiltrating immune cells, they are also most highly expressed in a subtype associated with immunosuppression. This may be attributed to the potential influence of specific infiltrating cells, such as mast cells and M2 macrophages, contributing to the establishment of an immunosuppressive tumor microenvironment [35659268], although further experiments are required to test this hypothesis. This suggests that the role of F3, PLAT and C1S in cancer immunology may be more complex than initially assumed.

Identification of novel tumor targets provides alternative therapeutic approaches for clinical treatment [30, 31]. For example, regarding overexpression of bromodomain containing (BRD4) and c-Myc in melanoma and hepatocellular carcinoma, thus, ARV-825, a proteolysis targeting chimera that specifically targets BRD4 and c-Myc was designed for anticancer therapy [32–34]. Given the profound and detrimental prognostic impact of F3, PLAT, and C1S, coupled with their elevated expression in tumor tissues, targeting these genes or their associated pathways may represent a promising therapeutic strategy. We investigated the potential correlation between VRG levels and drug sensitivity in different cancer cell lines [35]. Our results indicate that drugs, such as ciclosporin, ouabain and 6 − mercaptopurine exhibit negative half-maximal inhibitory concentration (IC50) values with F3, PLAT and C1S, respectively, indicating potential robust responses in tumors. Ciclosporin, also known as cyclosporine or cyclosporine A, primarily known for immunosuppression, is primarily recognized for its immunosuppressive properties. However, ongoing research is investigating its potential as a chemosensitizer or immunomodulator in cancer therapy [36]. Ouabain, a specific Na + /K + -ATPase inhibitor, disrupts cancer metabolism and effects immune responses [37, 38]. However, the precise mechanisms by which ouabain influences metabolism and modulates immune responses remains elusive. 6 − mercaptopurine is widely used in leukemia treatment and as an immunosuppressive agent [39]. As a pro-drug, 6 − mercaptopurin undergoes extensive intracellular metabolism, ultimately forming 6-thioguanine nucleotides, which leads to DNA strand breaks and apoptosis. However, the complex metabolic processes give rise to wide inter-individual variability in the systemic exposure to 6 − mercaptopurine [39]. In our study, we found that patients with elevated VRG expression display abnormal immune activity. Given that all three drugs possess immunosuppressive properties, this finding strengthens their potential efficacy in treating patients with high VRG expression. However, the potential off-target effects on normal physiological processes and the potential for resistance mechanisms need to be take a consideration.

Nevertheless, our research has some limitations. Firstly, this study primarily relies on bioinformatic analyses to explore the VRG landscape. Though we employed multiplex immunofluorescence staining to examine the expression of F3, PLAT, and C1S across various cancer types, additional experimental evidence is required to substantiate the observed cellular and molecular associations in the future and to fully understand the therapeutic benefits and limitations of utilizing ciclosporin, ouabain, and 6-mercaptopurine in cancer treatment. Secondly, though this study identifies significant associations between VRGs and the immune microenvironment, it was unable to conclusively determine whether VRGs influence the patient prognosis through immune infiltration, thus warranting further investigation underlying this relationship. Moreover, further exploration of gene therapy interventions tailored to the specific molecular signatures of upregulated VRGs in cancer could holds the potential to pave the way for innovative therapeutic strategies.

In conclusion, our comprehensive study establishes the expression profiles of VRGs across pan-cancer, revealing their association with cancer prognosis, correlation with TME, and stemness score, particularly in GBMLGG. Furthermore, the expression levels of F3, PLAT and C1S in tumor cells are linked to sensitivity to specific drugs. These findings underscore the clinical relevance of VRGs as predictors and therapeutic targets for personalized cancer therapy, serving as vital references for future research endeavors.

## Methods

### Selection of VRGs

To identify genes related to VTE, we retrieved information on complement and coagulation pathways from the MSigDB (Molecular Signatures; https://www.gsea-msigdb.org/gsea/msigdb/) and KEGG (kyoto encyclopedia of genes and genomes; hsa04610; https://www.genome.jp/kegg/) databases. To ensure consistency, we overlapped the genes from these pathways and ultimately identified a total of 21 candidate genes. To further refine our selection, we constructed a protein–protein interaction (PPI) network using STRING (Search Tool for the Retrieval of Interacting Genes; https://string-db.org/) [40] and visualized it with Cytoscape v3.91 [41]. The cytoHubba, a plug-in of Cytoscape, was utilized to extract the hub genes using the MNC algorithm.

### VRG expression and prognosis value analysis

The mRNA expression levels of VRGs and clinical datasets were extracted from the TCGA database (https://portal.gdc.cancer.gov). Details of the numbers of samples used are provided in Table S1. The mRNA expression of VRGs was compared across different cancers, and the differences in expression between cancer and paired para-cancer tissues were analyzed. The Xiantao tool (https://www.xiantao.love) was used to analyze and visualize prognosis values including OS, DSS, DFI and PFI. The GEO expression matrix file (GSE18491, http://www.ncbi.nlm.nih.gov/geo/) was employed in the analysis to generate Kaplan–Meier (K-M) survival curves.

### Multiplex immunofluorescence staining

Multiple cancer samples and their corresponding adjacent tissues for 91 patients were obtained from Outdo Biotech (HOrgC180PG01-1, Shanghai, China). Multiplex immunofluorescence staining was conducted as previously description [42]. Briefly, after deparaffinization, rehydration, antigen retrieval and protein blocking, the tissue array slide was then stained with the following primary antibodies including anti-C1S (Rabbit, 1:250 dilution, Affinity Biosciences, Jiangsu, China), anti-F3 (Rabbit, 1:100 dilution, Affinity Biosciences), and anti-PLAT (Rabbit, 1:100 dilution, Affinity Biosciences) sequentially. After washing with TBST buffer, the slide was incubated with an HRP-conjugated secondary antibody for 10 min, followed by addition of fluorophore diluted with tyramide signal amplification (TSA) at a 1:200 dilution (Panovue, Beijing) for an additional 10 min. The stained-slide was counterstained with 4’,6-Diamidino2-phenylindole dihydrochloride (DAPI) to indicate the nuclei. Finally, the stained images were captured using the SlideViewer v2.5 software (Hamamatsu Photonics Corp, Japan).

### Drug sensitivity analysis

The expression data and drug sensitivity data were acquired from the CellMiner (http://discover.nci.nih.gov/cellminer) [35], a free online tool for studying molecular characteristics and pharmacology. Data were processed using the “impute” and “limma” R packages. Finally, the data were visually depicted using the “ggplot2” and “ggpubr” R packages.

### Supplementary Information

Additional file 1: Supplementary Figures.

Additional file 2: Table S1.

## Data Availability

All data generated or analyzed during this study are included in this published article.
