# Supplementary material for: Pan-cancer analysis identifies venous thromboembolism-related genes F3, PLAT, and C1S as potential prognostic biomarkers for glioblastoma and lower grade glioma
Source: Mol Biomed. 2024 Aug 24;5:34. doi: 10.1186/s43556-024-00197-9 (PMC11343955; doi:10.1186/s43556-024-00197-9)
Supplement: Supplementary file 1 — Additional file 1: Supplementary Figures. [file 43556_2024_197_MOESM1_ESM.docx]

**Pan-cancer analysis identifies venous thromboembolism-related genes F3, PLAT, and C1S as potential prognostic biomarkers for glioblastoma and lower grade glioma**

Jing Zhang^1, 2*, #^, Qian Zhao^2, #^, Yun Du^3, #^, Wannan Wang^1, 2^, Cuiqing Liu^4, *^

^1^Department of Radiology, The First Affiliated Hospital of Jinan University, Guangzhou 510630, China;

^2^MOE Key Laboratory of Tumor Molecular Biology and Key Laboratory of Functional Protein Research of Guangdong Higher Education Institutes, Institute of Life and Health Engineering, College of Life Science and Technology, Jinan University, Guangzhou 510632, China;

^3^ Department of Nursing, The First Affiliated Hospital of Jinan University, Guangzhou 510630, China.

^4^Department of Surgery, The First Affiliated Hospital of Jinan University, Guangzhou 510630, China.

^#^These authors contributed equally to this work.

***Corresponden****ce author:**

Jing Zhang

Department of Radiology, The First Affiliated Hospital of Jinan University, Guangzhou 510630, China; E-mail: zj6410@jnu.edu.cn

Cuiqing Liu

Department of Surgery, The First Affiliated Hospital of Jinan University, Guangzhou 510630, China. E-mail address: 13302381081@126.com

**Supplementary Methods**

**Gene Ontology (GO)and KEGG enrichment analysis**

GO and KEGG enrichment was conducted using the method previously described [[1](#_ENREF_1)]. Briefly, we employed the R package "clusterProfiler v4.4.4" [[2](#_ENREF_2)] to perform cellular component (CC), biological pathway (BP), molecular function (MF), and KEGG enrichment analyses for VRGS.

**Immune‑related analysis**

The Stromal, Immune, and ESTIMATE scores were analyzed using the Xiantao online tool. The immune subtypes were analyzed using the TISIDB database (<http://cis.hku.hk/TISIDB/index.php>) [[3](#_ENREF_3)]. The infiltration scores of 22 types of immune cells in each tumor patient were examined through the online tool Sangerbox (<http://sangerbox.com>) [[4](#_ENREF_4)].

**Genetic variations analysis**

Genetic variations of VRGs, including mutations, structural variants, amplification, deep deletion, and multiple alterations, were identified using the CBioPortal tool (<http://cbioportal.org>) [[5](#_ENREF_5)]. The deletion/amplification statistics of heterozygous/homozygous copy number variations (CNV) of VRGs across various cancer types were investigated using the Genome Cancer Analysis (GSCA) (<http://bioinfo.life.hust.edu.cn/GSCA>) [[6](#_ENREF_6)] and visualized using a pie chart.

**Methylation analysis**

The methylation differences between paracancer and cancer samples of VRGs across different cancers were analyzed using the online tool GSCA. Subsequently, the table presenting detailed information about the methylation difference was downloaded, and the data were visually depicted using the R software v4.2.1 "ggplot2 v3.3.6" package.

**Correlation analysis of VRGs expression and stemness scores, TMB and MSI levels**

DNA stemness score (DNAss), for each tumor were acquired from a previous study [[7](#_ENREF_7)] using Sangerbox. TMB of each tumor was calculated based on the dataset for simple nucleotide variation in TCGA samples acquired from the GDC database Genomic Data Commons; https://portal.gdc.cancer.gov), utilizing the “maftools v2.8.05” R package with the TMB function. MSI for each cancer patient was collected from previous study [[8](#_ENREF_8)], using the online tool Sangerbox. Pearson correlation analysis was performed to analyze the correlation between these data and VRGs expression. Bubble plots or radar plots were generated using Sangerbox to present the results of the correlation analysis.

**References**

1. Huang Z, Zhu S, Han Z, Li C, Liang J, Wang Y et al. Proteome-Wide Analysis Reveals TFEB Targets for Establishment of a Prognostic Signature to Predict Clinical Outcomes of Colorectal Cancer. Cancers. 2023;15(3). https://doi.org/10.3390/cancers15030744.

2. Yu G, Wang LG, Han Y, He QY. clusterProfiler: an R package for comparing biological themes among gene clusters. OMICS. 2012;16(5):284-7. https://doi.org/10.1089/omi.2011.0118.

3. Ru B, Wong CN, Tong Y, Zhong JY, Zhong SSW, Wu WC et al. TISIDB: an integrated repository portal for tumor-immune system interactions. Bioinformatics. 2019;35(20):4200-2. https://doi.org/10.1093/bioinformatics/btz210.

4. Shen W, Song Z, Zhong X, Huang M, Shen D, Gao P et al. Sangerbox: A comprehensive, interaction-friendly clinical bioinformatics analysis platform. iMeta. 2022;1(3):e36. https://doi.org/10.1002/imt2.36.

5. Cerami E, Gao J, Dogrusoz U, Gross BE, Sumer SO, Aksoy BA et al. The cBio cancer genomics portal: an open platform for exploring multidimensional cancer genomics data. Cancer discovery. 2012;2(5):401-4. https://doi.org/10.1158/2159-8290.CD-12-0095.

6. Liu CJ, Hu FF, Xia MX, Han L, Zhang Q, Guo AY. GSCALite: a web server for gene set cancer analysis. Bioinformatics. 2018;34(21):3771-2. https://doi.org/10.1093/bioinformatics/bty411.

7. Malta TM, Sokolov A, Gentles AJ, Burzykowski T, Poisson L, Weinstein JN et al. Machine Learning Identifies Stemness Features Associated with Oncogenic Dedifferentiation. Cell. 2018;173(2):338-54 e15. https://doi.org/10.1016/j.cell.2018.03.034.

8. Bonneville R, Krook MA, Kautto EA, Miya J, Wing MR, Chen HZ et al. Landscape of Microsatellite Instability Across 39 Cancer Types. JCO Precis Oncol. 2017;2017. https://doi.org/10.1200/PO.17.00073.

**Supplementary Figures**


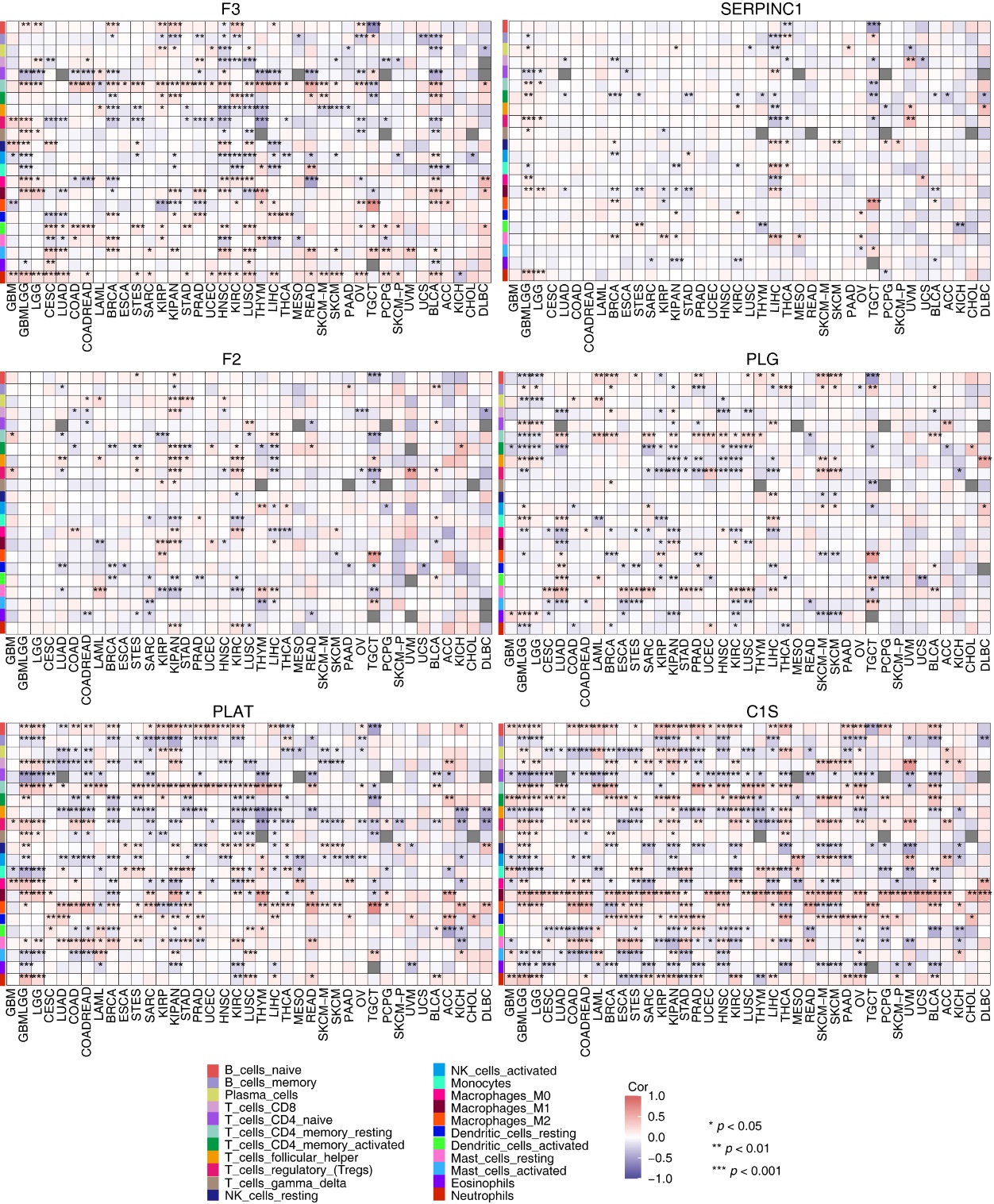


**Fig S1. Immune cell infiltration influenced by VRGs.** Correlation between 22 major types of immune cells and the expression of *F3*, *SERPINC1*, *F2*, *PLG*, *PLAT*, and *C1S*. **p* < 0.05, ***p* < 0.01, ****p* < 0.001


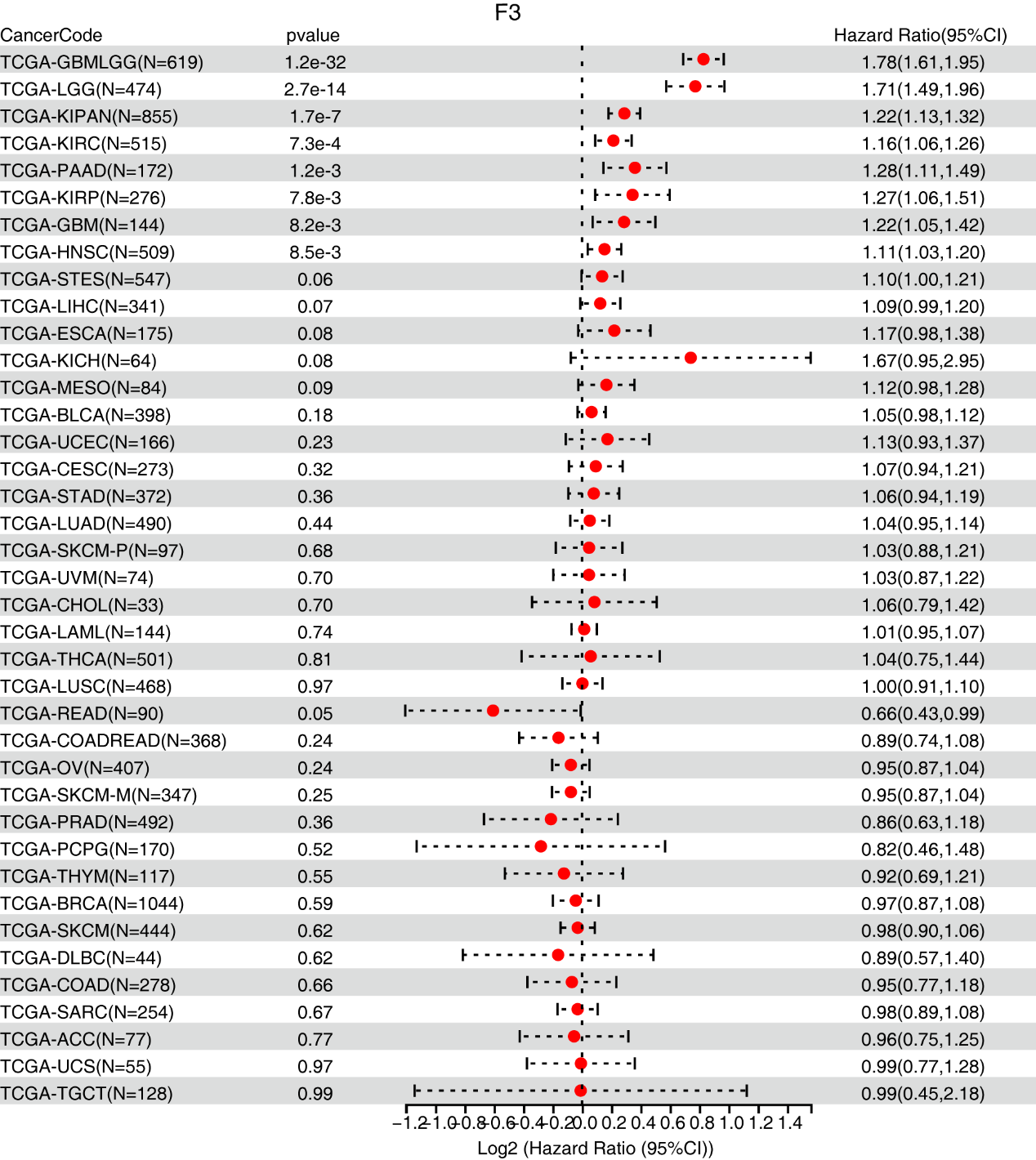


**Fig S2** Overall survival prognosis forest map of F3 in TCGA


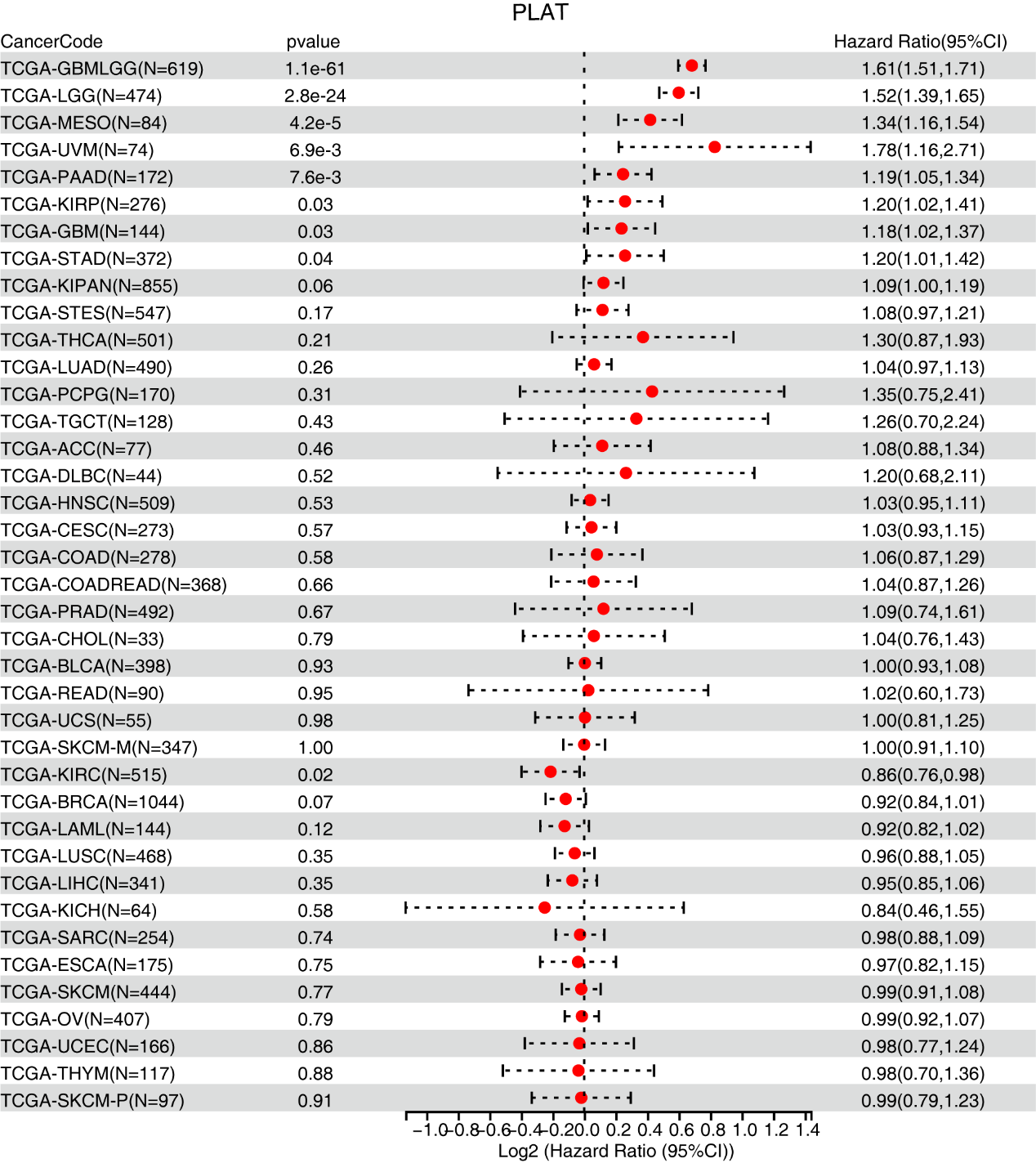


**Fig S3.** Overall survival prognosis forest map of PLAT in TCGA


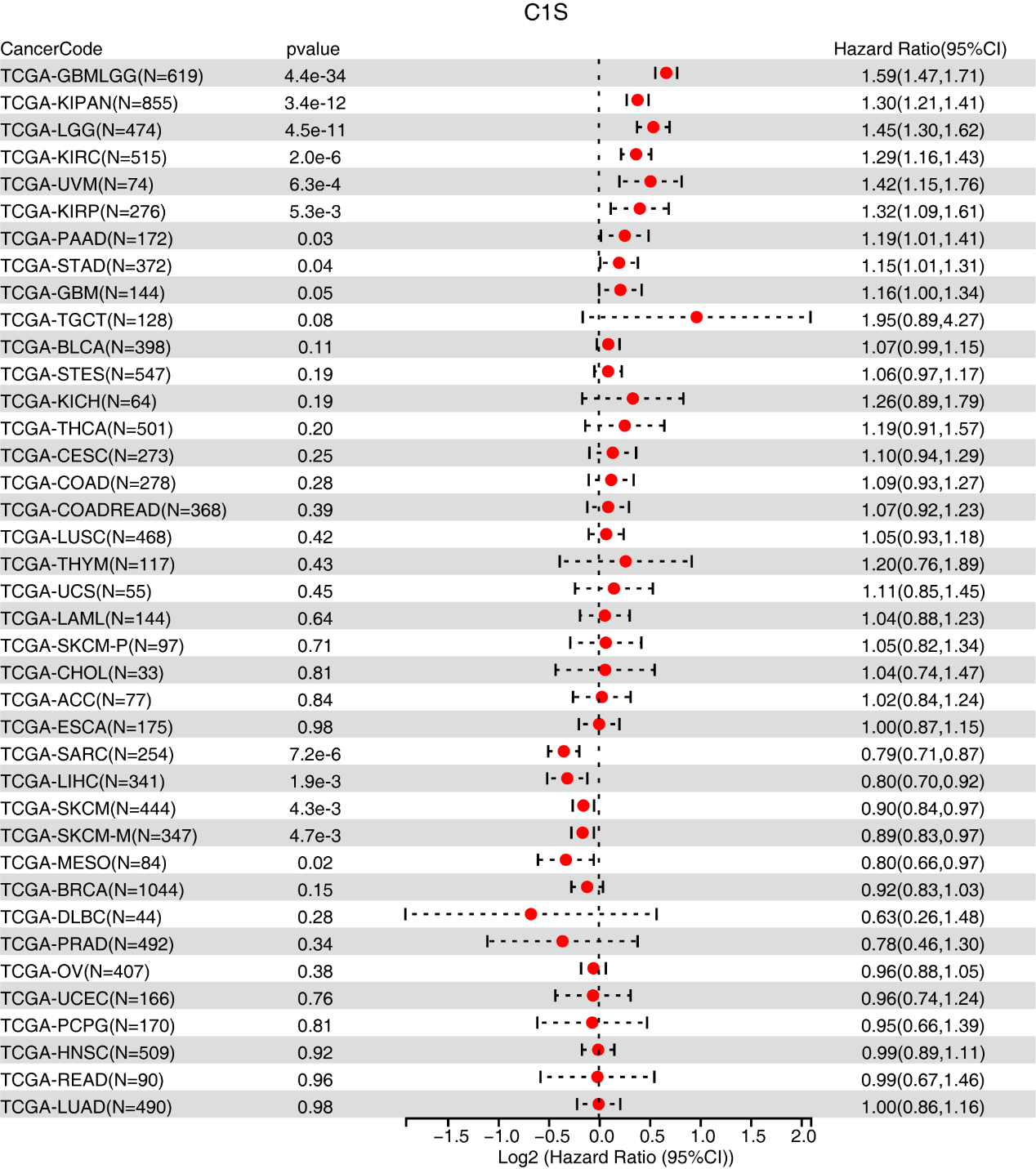


**Fig S4.** Overall survival prognosis forest map of C1S in TCGA


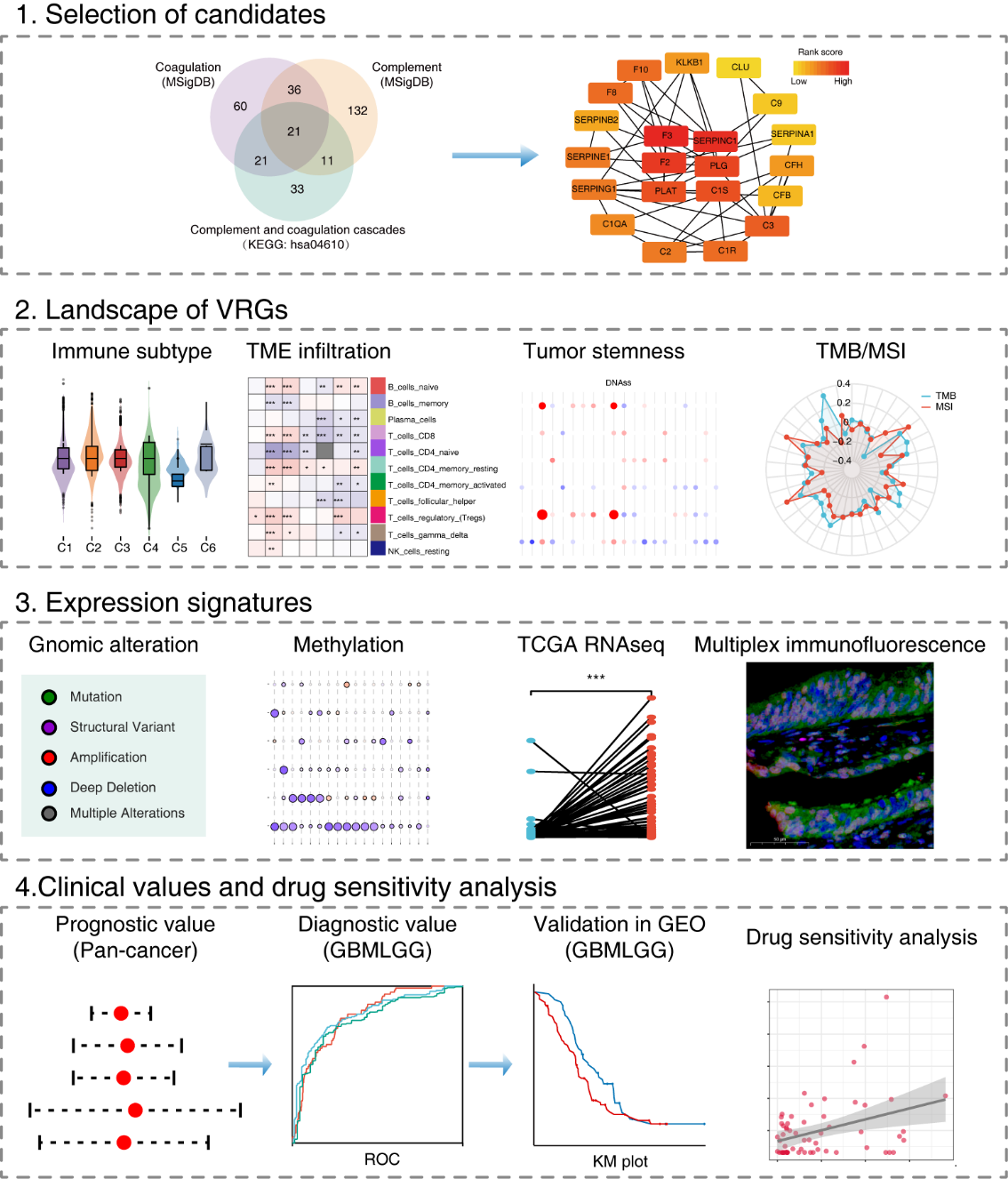


**Fig S5. Overview of the study design.**

**Supplementary Table**

**Table S1.** The number of samples used in each figure.

Provided separately, attached as Excel files.
